# Frontal and temporal lobe contributions to emotional enhancement of memory in behavioral-variant frontotemporal dementia and Alzheimer's disease

**DOI:** 10.3389/fnbeh.2014.00225

**Published:** 2014-06-24

**Authors:** Fiona Kumfor, Muireann Irish, John R. Hodges, Olivier Piguet

**Affiliations:** ^1^Neuroscience Research Australia, RandwickSydney, NSW, Australia; ^2^School of Medical Sciences, The University of New South WalesSydney, NSW, Australia; ^3^ARC Centre of Excellence in Cognition and its DisordersSydney, NSW, Australia; ^4^School of Psychology, The University of New South WalesSydney, NSW, Australia

**Keywords:** emotion, episodic memory, dementia, hippocampus, amygdala

## Abstract

Emotional events gain special priority in how they are remembered, with emotionally arousing events typically recalled more vividly and with greater confidence than non-emotional events. In dementia, memory and emotion processing are affected to varying degrees, however, whether emotional enhancement of memory for complex ecologically-valid events is differentially affected across dementia syndromes remains unclear, with previous studies examining effects of emotion on simple visual recognition only. Here, we examined memory for an emotionally arousing short story and a closely matched, emotionally neutral story in behavioral-variant frontotemporal dementia (bvFTD) (*n* = 13) and Alzheimer's disease (AD) (*n* = 14), and contrasted their performance with healthy controls (*n* = 12). Multiple-choice recognition memory for specific details of the story was assessed after a 1-h delay. While AD and control groups showed enhanced memory for the emotional story, the bvFTD group recalled a similar number of details from the emotional and neutral stories. Voxel-based morphometry analyses revealed emotional enhancement of memory correlated with distinct brain regions in each patient group. In AD, emotional enhancement was associated with integrity of the bilateral hippocampus, parahippocampal gyri, temporal fusiform gyrus and frontal pole, regions typically implicated in memory processes. In contrast in bvFTD, integrity of emotion processing regions, including the orbitofrontal cortex, right amygdala and right insula, correlated with the extent emotion enhanced memory. Our results reveal that integrity of frontal and temporal regions determine the quality and nature of emotional memories. While emotional enhancement of memory is present in mild AD, in bvFTD emotion does not facilitate memory retrieval for complex realistic events. This attenuation of emotional enhancement is due to degradation of emotion processing regions, which may be important for modulating levels of arousal in response to emotional events in these patients.

## Introduction

Events that are imbued with emotion are typically remembered more vividly and with more confidence than non-emotional events. This effect is thought to depend on interactions between frontal and temporal lobe structures. These regions are disproportionately affected in behavioral-variant frontotemporal dementia (bvFTD) and Alzheimer's disease (AD), the two most common younger-onset dementia disorders (Ratnavalli et al., 2002). These different patterns of neurodegeneration manifest as distinct clinical profiles. Emotion processing is impaired in bvFTD, whereas in AD emotion processing is relatively intact early in the disease process (Lavenu et al., 1999; Kumfor and Piguet, 2013; Kumfor et al., 2013a). For memory, the reverse pattern may be observed. AD patients show profound deficits in episodic memory due to impairments in storage, characterized by rapid forgetting, whereas, episodic memory performance is more variable in bvFTD (Pasquier et al., 2001; Graham et al., 2005; Hornberger et al., 2010, 2012; Hornberger and Piguet, 2012; Irish et al., 2013). Given their divergent clinical profiles, these patient groups provide an opportunity to examine emotional enhancement of memory and test how capacity for detection of emotional signals and memory storage impact on this complex cognitive ability.

Surprisingly, only one study has examined emotional enhancement of memory in bvFTD in comparison with AD to date (Kumfor et al., 2013b). The results revealed that bvFTD showed attenuation of the emotional enhancement effect, which was associated with atrophy in the orbitofrontal cortex, suggesting that the emotion processing deficits seen in bvFTD impact on their capacity for emotional enhancement of memory. The previous study, however, assessed simple visual recognition memory only, and did not examine whether memory for more complex ecologically valid, emotional events is also affected. Therefore, it remains to be seen whether emotion also fails to enhance memory for specific details of complex events in patients with dementia, which more closely reflect the types of events these patients experience in day-to-day life. In addition, the extent that other brain regions may contribute to emotional enhancement of memory in these patients, such as the amygdala and hippocampus, is unknown.

In AD, a larger number of studies have examined emotional enhancement of memory than in bvFTD, however the reported results have been highly variable. While some studies have demonstrated emotional enhancement of memory in these patients (Ikeda et al., 1998; Mori et al., 1999; Kazui et al., 2003), others report no effect (Kensinger et al., 2004). In the Kumfor et al. (2013b) study, a facilitatory effect of emotion in the AD group was observed, with these patients endorsing more emotional items as previously seen, compared to neutral items. Notably, AD patients generally showed a liberal response bias for recognizing emotional stimuli, also endorsing a high number of incorrect emotional items, suggesting that emotion may not directly improve memory *accuracy* in these patients. Variability in the type of task and the response requirements seems to influence the extent that emotion enhances memory in AD (Klein-Koerkamp et al., 2012). The few existing neuroimaging studies examining this effect in AD have reported that integrity of the amygdala, orbitofrontal cortex, and memory structures including the hippocampus and posterior cingulate cortex is related to the extent emotion enhances memory in these patients (Mori et al., 1999; Kumfor et al., 2013b; Landré et al., 2013).

Theoretical accounts of emotional enhancement of memory propose that the effect of emotion on memory varies, depending on whether the information is central or peripheral to the event. Existing evidence suggests that details that are central or intrinsic to the event are remembered at the expense of memory for peripheral details (the *trade-off* effect) (e.g., Kensinger et al., 2005, 2007). This enhanced memory for central event details is thought to be mediated by changes in attention (Kensinger, 2004), with emotion narrowing attention, so that more time is spent focusing on the central, emotional details, than other components of an event. In addition, emotional information is preferentially attended to, even when attentional resources are limited. This attention-mediated trade-off effect reportedly depends upon the integrity of similar structures as those involved in emotional memory more generally, especially the amygdala (Adolphs et al., 2001; Anderson and Phelps, 2001; Perrin et al., 2012). Thus, neurodegeneration of structures crucial for emotional enhancement of memory, such as the amygdala are also likely to influence the trade-off effect. To date, like emotional enhancement of memory itself; no studies have examined the trade-off effect in dementia, other than in AD. The limited information available suggests that memory for gist (at the expense of visual detail) for emotional stimuli may be preserved in AD (Perrin et al., 2012). Whether a trade-off effect between central and peripheral details is also observed in AD, however, remains unclear. Furthermore, no studies have investigated the trade-off effect in bvFTD to date.

The first aim of this study was to contrast emotional enhancement of memory in bvFTD and AD to determine the relative contributions of emotion and memory to this effect. It was hypothesized that bvFTD patients would show an attenuation of emotional enhancement of memory due to impaired emotion processing, whereas in AD, it was hypothesized that emotional enhancement of memory would be observed, despite an overall reduction in memory performance. The second aim was to investigate whether differences in the modulatory effect of emotion on central or peripheral details existed, and whether this effect differed across dementia syndromes. Finally, we aimed to identify the neural correlates involved in emotional enhancement of memory in these patient groups using voxel-based morphometry. We predicted that atrophy in frontal and temporal lobe structures would differentially contribute to memory and emotional enhancement of memory, and that common and distinct brain regions would contribute to performance in bvFTD and AD.

## Methods

### Participants

Thirteen bvFTD and 14 AD participants were compared with 12 healthy controls. Participants were recruited from FRONTIER, the frontotemporal dementia research group in Sydney, Australia. All patients were diagnosed based on current consensus criteria (McKhann et al., 2011; Rascovsky et al., 2011). Patients with bvFTD typically presented with behavioral disinhibition, apathy, loss of sympathy or empathy, perseverative behavior, change in food preferences and/or executive dysfunction, whereas AD patients showed deficits in episodic memory including learning and recall of recent information, together with mild reductions in naming, visuospatial abilities and executive functioning. Control participants were recruited from local community clubs. Exclusion criteria included: history of psychiatric or neurological conditions, centrally-acting medication, and limited proficiency in English. In addition, all controls were required to score >88/100 on the Addenbrooke's Cognitive Examination-Revised general screening measure of cognition (Mioshi et al., 2006). All participants or their Person Responsible provided informed consent according to the Declaration of Helsinki. The South Eastern Sydney Local Health District and the University of New South Wales ethics committees approved the study.

### General cognitive assessment

All participants were tested on neuropsychological measures of attention (Digit Span Forwards, maximum span, Wechsler, 1997; Trail Making Test A, Tombaugh, 2004), episodic memory (Doors Memory Test, Part A, Baddeley et al., 1994; Rey Complex Figure 3-min recall Meyers and Meyers, 1995; Rey Auditory Learning Verbal Test (RAVLT) 30-min recall and recognition Schmidt, 1996), language (Sydney Language Battery, Savage et al., 2013), executive functioning (Trail Making Test B, Tombaugh, 2004; Letter fluency, Spreen and Strauss, 1998) and emotion (Ekman 60, Young et al., 2002).

### Emotional memory task

To examine the effect of emotion on episodic memory we used a story task based on a well-validated paradigm that has been previously administered in healthy participants, and participants with amygdala lesions, and produced reliable findings (Heuer and Reisberg, 1990; Cahill and McGaugh, 1995; Cahill et al., 1995; Adolphs et al., 1997). In brief, participants were shown a narrated story, which was either emotionally arousing or emotionally neutral. Eleven slides were presented in total, and the story was divided into three phases. Phase 1 and Phase 3 were identical for both versions of the story, with the narration for Phase 2 differing in its emotional content across the two stories (Table 1). Participants were instructed to pay close attention to the pictures and the story, but were not informed that their memory for the story would be tested later. Immediately following the presentation of the story, participants were asked to rate the story for understanding (/10) and emotionality (/10).

**Table 1:**
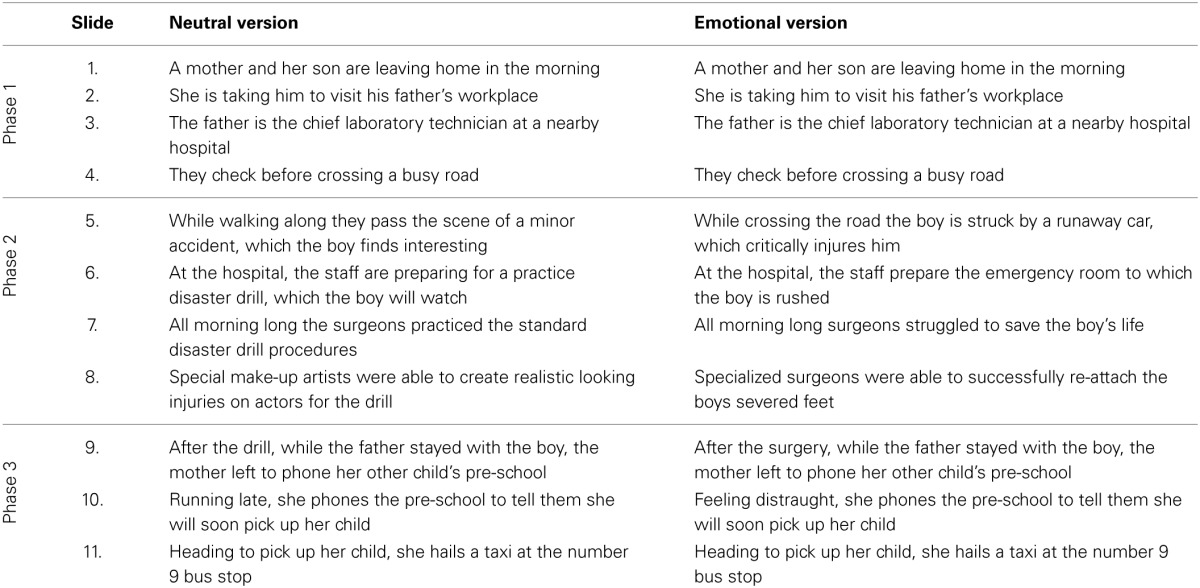
**Narratives for the neutral and emotional versions of emotional memory task based on Cahill and McGaugh (1995)**.

Following a 1-h delay, filled with unrelated neuropsychological tests, episodic memory for the story was assessed using a detailed, well-established multiple-choice questionnaire, 76 questions in total. Between 5 and 9 questions were asked per slide. The questions have been previously classified as central or peripheral to the story allowing for investigation of the following components of memory: (i) combined (central and peripheral) memory (ii) memory for central details, and (iii) memory for peripheral details. Questions classified as central assessed memory for details directly relevant to the plot of the story (e.g., the boy's legs were re-attached), whereas peripheral details assessed memory for information that could be altered without changing the story (e.g., the boy was seen lying on his back).

Two weeks later, participants were tested on the alternate version of the story. The order of story presentation was counterbalanced across participants.

### Image acquisition

Participants underwent whole brain structural magnetic resonance imaging (MRI) with a 3-Tesla (3-T) Phillips MRI scanner. High resolution coronal plane T1-images were obtained using the following protocol: 256 × 256, 200 slices, 1 mm^2^ in-plane resolution, 1 mm slice thickness, echo time/ repetition time = 2.6/ 5.8 ms, flip angle α = 19°. Brain scans were available for 13 bvFTD, 14 AD, and 10 healthy controls. One control could not be scanned due to MRI contraindications and MRI data for another control was not available because of technical difficulties.

### Data preprocessing

FSL voxel-based-morphometry, part of the FMRIB software library package (http://www.fmrib.ox.ac.uk/fsl/fslvbm/index.html; Smith et al., 2004) was used to analyse the MRI data (Ashburner and Friston, 2000; Mechelli et al., 2005; Woolrich et al., 2009). Structural images were brain-extracted using BET, then tissue segmentation was conducted with automatic segmentation (FAST) (Zhang et al., 2001). Gray matter partial volume maps were aligned to Montreal Neurological Institute standard space (MNI152) using non-linear registration (FNIRT) (Andersson et al., 2007a,b), which uses a *b*-spline representation of the registration warp field (Rueckert et al., 1999). A study-specific template was created and the native gray matter images were non-linearly re-registered. Modulation of the registered partial volume maps was carried out by dividing them by the Jacobian of the warp field, and the modulated, segmented images were smoothed with an isotropic Gaussian kernel with a sigma of 3 mm.

### Statistical analyses

Data were analyzed using IBM Statistics SPSS Version 20. Kolmogorov-Smirnov tests were run to check suitability of variables for parametric analysis. For general cognitive screening and demographic variables multivariate analyses of variance (ANOVA), with *post-hoc* analyses were conducted using Sidak correction for multiple comparisons to explore between group differences.

Subjective ratings of understanding and emotionality of the two stories were investigated using repeated measures ANOVA with version (Neutral, Emotional) as the within subjects variable, and diagnosis (bvFTD, AD, controls) as the between subjects variable. *Post-hoc* analyses were also conducted to investigate main and interaction effects, with Sidak correction for multiple comparisons.

For the analyses on the emotional memory task, performance from each story was first divided into the three phases (Phase 1: Slides 1-4; Phase 2: Slides 5-8; Phase 3: Slides 9-11, Table 1). For each phase of the emotional and neutral story, the following scores were calculated: (i) percent correct combined (central & peripheral) score, (ii) percent correct central score and (iii) percent correct peripheral score. Next, three repeated measures ANOVAs were conducted examining combined, central and peripheral memory with version (Neutral, Emotional) and Phase (1, 2, 3) as the within subjects variables and diagnosis (bvFTD, AD, controls) as the between subjects variable. *Post-hoc* analyses using Sidak correction for multiple comparisons were conducted to investigate main and interaction effects.

### Voxel-based morphometry analyses

A voxel-wise general linear model was applied to investigate gray matter intensity differences, using permutation-based, non-parametric statistics, with 5000 permutations per contrast (Nichols and Holmes, 2002). Differences in gray matter intensity between bvFTD, AD and controls was assessed using *t*-tests (Figure S1).

Next, correlations between memory performance and gray matter intensity were conducted. The combined scores for the emotional and neutral stories were entered simultaneously into the design matrix. Contrasts investigated correlations averaged across emotional and neutral stories [1,1] and correlations for emotional story memory, taking into account neutral story memory performance [1,0] (Kumfor et al., 2013b). Correlations between recognition performance and gray matter intensity were investigated combining all participants (bvFTD, AD, controls). Next, the same approach was used to identify associations with gray matter intensity and central memory and peripheral memory respectively. Finally, to identify neural correlates of emotional memory specific to each patient group, combined memory performance was investigated using the same analyses described above, in each patient group combined with controls. This approach has been shown to achieve greater variance in scores, increasing the statistical power to detect behavioral correlations (Sollberger et al., 2009; Irish et al., 2012). For all analyses, the statistical threshold was set at *p* < 0.005 uncorrected for multiple comparisons, consistent with previous studies (Rameson et al., 2011; Visser and Lambon Ralph, 2011). In addition, a conservative cluster extent threshold of 100 voxels was used to reduce the likelihood of false positives. This approach aims to minimize Type I errors, while also balancing the potential risk of Type II error (Lieberman and Cunningham, 2009).

Anatomical locations of significant results were overlaid on the Montreal Neurological Institute (MNI) standard brain, with maximum coordinates provided in MNI stereotaxic space. Anatomical labels were determined with reference to the Harvard-Oxford probabilistic cortical and subcortical atlases.

## Results

### Demographics

All groups were matched for age [*F*_(2, 38)_ = 1.254; *p* = 0.298], however the difference in level of education was significant [*F*_(2, 38)_ = 6.882; *p* = 0.003] with both the bvFTD (*p* = 0.014) and AD (*p* = 0.005) group having fewer years of education than controls. Patient groups were matched for years of education (*p* > 0.05). Chi-squared tests revealed no difference in sex distribution (*p* > 0.05) across participant groups. Patient groups were matched for disease duration (*p* > 0.05) (Table 2).

**Table 2:** **Demographic characteristics of the study samples**.

**Demographics**	**bvFTD *n* = 13**	**AD *n* = 14**	**Controls *n* = 12**	***F***	***Post-hoc***
Sex (M/F)	8/5	11/3	8/4	ns	
Age (years)	66.5 ± 9.4	69.1 ± 7.9	71.3 ± 5.0	ns	
Education (years)	11.1 ± 2.6	10.7 ± 3.5	14.9 ± 3.2	^*^	Patients < Con
Disease duration (months)	69.7 ± 37.3	62.5 ± 53.1	-	ns	

Values are: Mean ± Standard deviation. ns p > 0.05;

* p < 0.05.

### General cognitive performance

Neuropsychological test scores revealed profiles typical of each dementia syndrome (Table 3). In brief, the bvFTD group showed impairments in general cognition (ACE-R), attention (Digit Span Forwards), working memory (Digit Span Backwards; Trails B time) and fluency, consistent with their clinical presentation of inattention and executive dysfunction. In addition, deficits in episodic memory were seen on Doors A, Rey Complex Figure and RAVLT 30-min recall, but not recognition. On the visual memory measures, bvFTD performed as poorly as the AD group, whereas on the verbal memory measure, bvFTD scored lower than controls but were not impaired to the same level as the AD group. Consistent with previous findings, the bvFTD group performed worse than controls and the AD group on the Ekman 60 task, indicative of severe facial emotion recognition impairment in these patients.

**Table 3:** **Performance on neuropsychological tests for the study samples**.

**Cognitive test**	**bvFTD**	**AD**	**Controls**	***F***	***Post-hoc* test**
ACE-R (100)	79.3 ± 12.7	77.4 ± 7.4	94.9 ± 3.9	^**^	Patients < Con
Digits-F	5.6 ± 1.2	5.6 ± 1.3	7.6 ± 1.1	^**^	Patients < Con
Digits-B	3.8 ± 1.0	3.8 ± 0.7	5.8 ± 1.1	^**^	Patients < Con
Trails A (sec)^a^^,^^e^	58.3 ± 36.9	47.9 ± 22.1	34.3 ± 9.2	ns	
Trails B (sec)^d^^,^^f^	212.8 ± 91.1	162.5 ± 72.6	82.0 ± 27.0	^**^	Patients > Con
Naming (30)	23.1 ± 4.3	21.9 ± 3.5	26.3 ± 2.4	^*^	AD < Con
Doors A (12)^b^^,^^e^	8.6 ± 1.6	7.2 ± 2.4	10.9 ± 1.0	^**^	Patients < Con
RCF Copy (36)^e^	27.7 ± 5.4	29.7 ± 3.6	30.9 ± 3.3	ns	
RCF 3-min Delay (36)^e^	10.8 ± 6.9	6.5 ± 5.3	18.1 ± 5.9	^**^	Patients < Con
RAVLT 30-min recall (15)^a^^,^^g^	5.0 ± 4.0	1.1 ± 1.5	11.9 ± 2.1	^**^	Patients < Con; AD < bvFTD
RAVLT 30-min recognition (15)^c^^,^^g^	10.8 ± 4.8	10.5 ± 3.2	14.1 ± 1.4	^*^	AD < Con
Fluency^a^^,^^e^	21.4 ± 10.8	28.6 ± 12.2	53.2 ± 13.4	^**^	Patients < Con
Ekman 60 (60)^e^	36.0 ± 13.3	41.9 ± 6.2	50.2 ± 4.5	^**^	bvFTD < Con

Values are: Mean ± Standard deviation. Maximum scores on cognitive tests in parentheses. Abbreviations: ns = p > 0.05;

* p < 0.05;

** p < 0.001. Con, controls; ACE-R, Addenbrooke's Cognitive Examination-Revised; Digits-F, Digit Span forwards, maximum span; Digits-B, Digit Span backwards, maximum span; RAVLT, Rey Auditory Verbal Learning Test; RCF, Rey Complex Figure. Missing data: bvFTD missing

a 1;

b 2,

c 3,

d 4; AD missing:

e 1,

f 2,

g 3.

In contrast, AD patients were impaired on the general cognitive screening measure (ACE-R) and showed deficits in attention (Digit Span Forwards), working memory (Digit Span Backwards; Trails B) and marked anomia, performing below bvFTD and controls on the confrontation naming task. Consistent with their clinical presentation, episodic memory was severely impaired, with AD patients performing below controls on all episodic memory tasks (Doors A, Rey Complex Figure, RAVLT recall and recognition) and showing impaired performance compared to bvFTD on the RAVLT recall. Fluency was also reduced compared to controls. Importantly, AD performance on the Ekman 60 task was relatively preserved (Table 3).

### Emotional memory task

#### Subjective ratings of understanding

**Figure 1:**
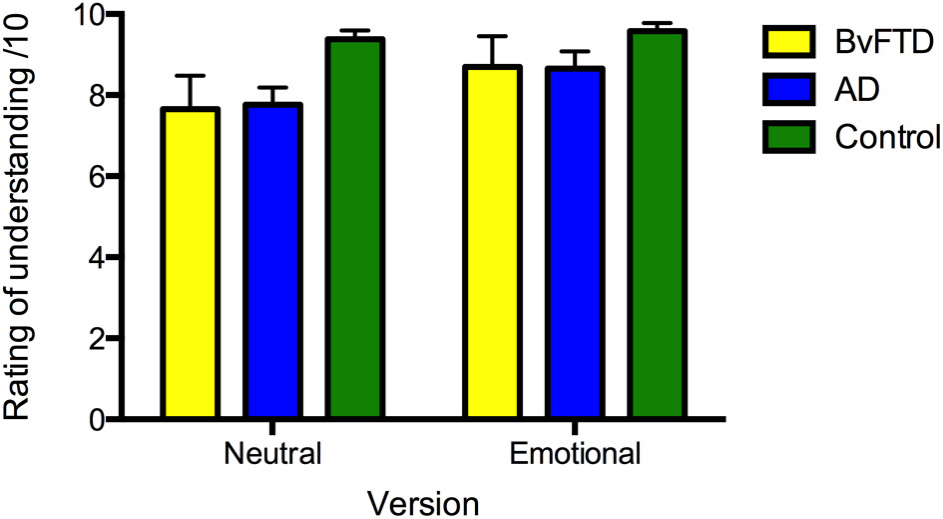
**Ratings of subjective level of understanding of the neutral and emotional versions of the story in: behavioral-variant frontotemporal dementia (bvFTD), Alzheimer's disease (AD), and controls**. Maximum rating is 10. Error bars represent standard error of the mean.

All groups rated the stories similarly for subjective level of understanding [*F*_(2, 36)_ = 2.207, *p* = 0.125] (see Figure 1). A main effect of version was significant [*F*_(1, 36)_ = 7.330, *p* = 0.010], indicating that all groups rated the emotional story as being better understood subjectively than the neutral version; nevertheless, the level of understanding was high for both stories. No version x diagnosis interaction was observed [*F*_(2, 36)_ = 0.904, *p* = 0.414], and between groups, the subjective ratings did not differ across story versions (all *p* > 0.05).

#### Subjective ratings of emotionality

**Figure 2:**
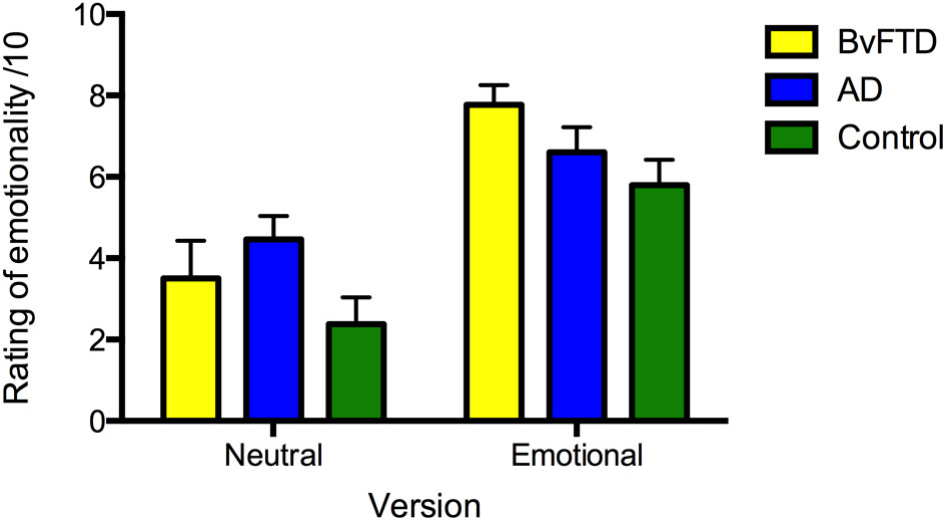
**Ratings of perceived emotionality of the neutral and emotional story versions in: behavioral-variant frontotemporal dementia (bvFTD), Alzheimer's disease (AD), and controls**. Maximum rating is 10. Error bars represent standard error of the mean.

For ratings of emotionality, the main effect of version was significant [*F*_(1, 36)_ = 65.710, *p* < 0.001], with the emotional story rated as more emotional than the neutral story (see Figure 2). Within group comparisons revealed that all groups rated the emotional story as more emotional than the neutral story (AD: *p* = 0.003; bvFTD: *p* < 0.001; control: *p* < 0.001). No main effect of diagnosis [*F*_(2, 36)_ = 2.295, *p* = 0.115] or interaction between diagnosis and version [*F*_(2, 36)_ = 2.442, *p* = 0.101] was observed.

#### Effect of emotion on combined (central and peripheral) memory

**Figure 3:**
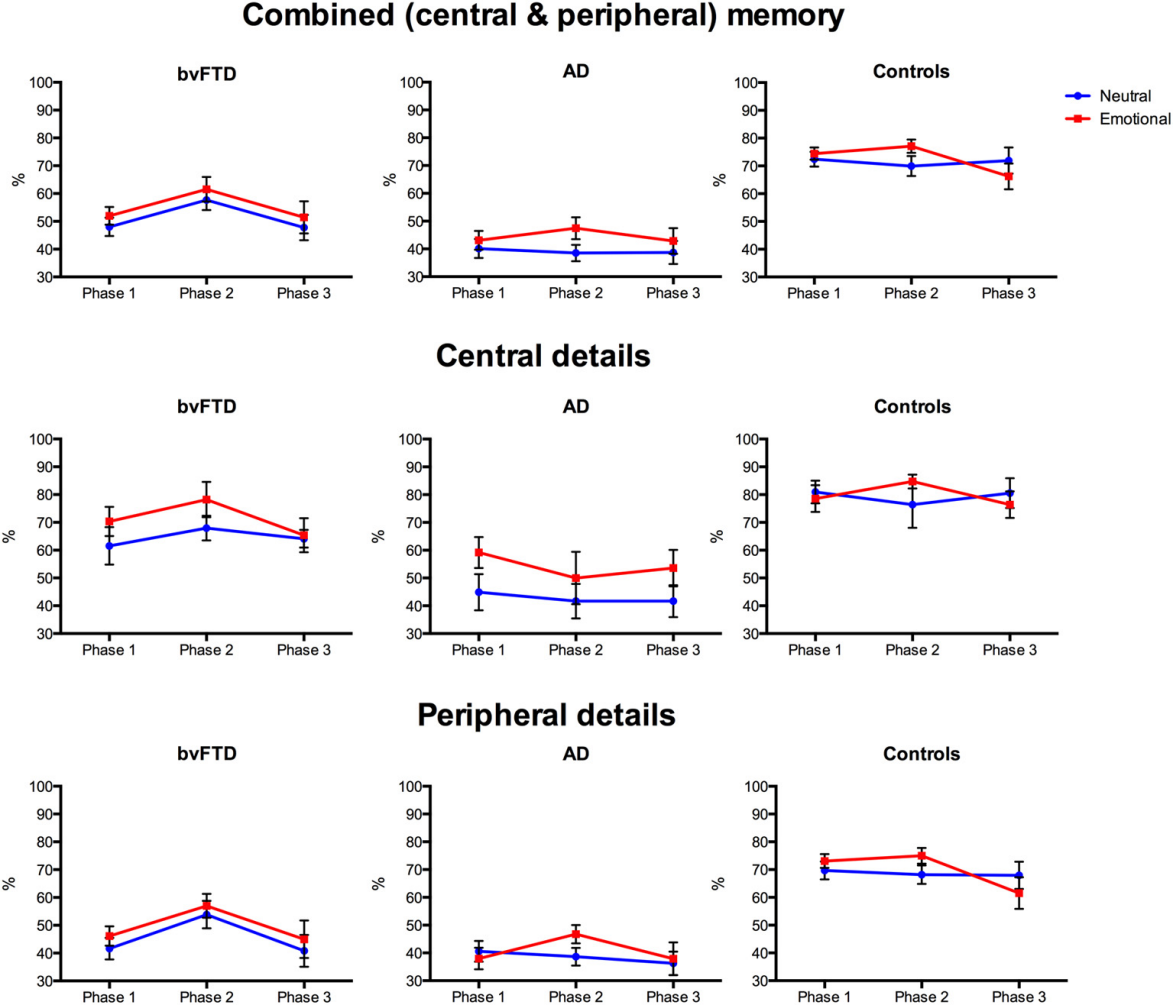
**Profiles of emotional memory performance in behavioral-variant frontotemporal dementia (bvFTD), Alzheimer's disease (AD), and controls**. Graphs show percent correct performance for the emotionally arousing (red) and neutral (blue) stories across the three story phases. Error bars represent standard error of the mean.

Investigation of memory performance revealed differential effects of emotion on combined memory scores, as shown in Figure 3. A main effect of diagnosis was significant [*F*_(2, 36)_ = 30.376, *p* < 0.001, η^2^_ρ_ = 0.628] with both the bvFTD (*p* < 0.001) and AD (*p* < 0.001) groups performing worse than controls and AD also showing worse memory than bvFTD (*p* = 0.016). A significant main effect of version emerged [*F*_(1, 36)_ = 4.871, *p* = 0.034, η^2^_ρ_ = 0.119], indicating that more details were remembered overall from the emotional than the neutral story. The main effect of phase was significant [*F*_(1, 52)_ = 4.456, *p* = 0.027, η^2^_ρ_ = 0.110], with more details recalled from Phase 2 than Phase 1 (*p* = 0.010), and a similar trend when compared to Phase 3 (*p* = 0.054). This effect was driven by the bvFTD group, which remembered more details from Phase 2 than Phase 1 (*p* < 0.001) and Phase 3 (*p* = 0.041), irrespective of emotional content. In contrast, no difference in memory performance across phases in the control and AD groups was observed, when averaged across story version (all *p* > 0.05). No significant interactions between phase, diagnosis and/or version were observed (all *p* > 0.05).

Given the main effect of diagnosis and the *a priori* hypothesis of a differential effect of emotion on memory across groups, planned contrasts were conducted to investigate differences within each group. In bvFTD, no difference in memory was seen between the neutral and emotional story on any Phase (Phase 1: *p* = 0.325; Phase 2: *p* = 0.259; Phase 3: *p* = 0.414). In contrast, the AD group showed enhanced memory for Phase 2 of the emotional story compared to the neutral story (*p* = 0.009), but no difference was observed for Phase 1 (*p* = 0.447) or Phase 3 (*p* = 0.337). Similarly, for controls, enhanced memory for the emotional story was seen for Phase 2 (*p* = 0.048) but not Phase 1 (*p* = 0.631) or Phase 3 (*p* = 0.222).

To summarize, the rating of emotional content of the two stories was similar across the three groups. For level of understanding, both the bvFTD and AD groups subjectively rated the emotional story as better understood than the neutral story. Importantly, however, memory for the two stories differed across groups. Combined memory scores were impaired in both patient groups compared to controls, with the AD group performing significantly below both control and bvFTD groups. Within group analyses revealed that AD and controls showed enhanced memory for Phase 2 of the emotional story compared to the neutral story. In contrast, no emotional enhancement of memory was seen in bvFTD, with a similar level of memory performance observed irrespective of emotional content.

#### Effect of emotion on central details

Examination of memory for central details, shown in Figure 3, revealed different profiles for each diagnostic group. An overall effect of diagnosis was again significant [*F*_(2, 36)_ = 15.792, *p* < 0.001, η^2^_ρ_ = 0.467], with the AD group performing significantly below control (*p* < 0.001) and bvFTD (*p* = 0.004) levels. In contrast, the bvFTD group remembered a similar number of central details as controls (*p* > 0.05). The overall effect of version was significant, with central details of the emotional story remembered better than the neutral story [*F*_(1, 36)_ = 6.929, *p* = 0.012, η^2^_ρ_ = 0.161]. This effect was driven by the AD group, who remembered more central details from the emotional than the neutral story (*p* = 0.006). Unexpectedly, within group analyses indicated that the difference in memory between the emotional and neutral story versions in the AD group was seen primarily in Phase 1 (*p* = 0.020), and Phase 3 (*p* = 0.053), rather than Phase 2 (*p* = 0.286). No difference between memory for the emotional and neutral stories was seen in control (*p* = 0.891) and bvFTD (*p* = 0.110) groups, averaged across phase or when each phase was examined separately (all *p* > 0.05). For central details, the main effect of phase [*F*_(2, 67)_ = 0.545, *p* = 0.571, η^2^_ρ_ = 0.015] was not significant and no significant interactions were observed between phase, diagnosis and version (*p* > 0.05).

In summary, the AD group remembered significantly fewer details compared with the bvFTD and control groups, demonstrating marked episodic memory deficits. In spite of this degradation of overall memory, emotion had an enhancing effect on central details, with the AD group showing greatest benefits, remembering significantly more details of the emotional than the neutral story. No difference in memory for central details according to emotional content was seen in bvFTD or controls.

#### Effect of emotion on peripheral details

For peripheral details, a significant main effect of diagnosis was observed [*F*_(2, 36)_ = 26.280, *p* < 0.001, η^2^_ρ_ = 0.593], with the AD (*p* < 0.001) and bvFTD (*p* < 0.001) groups performing significantly below controls, shown in Figure 3. No difference between patient groups was observed (*p* > 0.05). Although the main effect of version was not significant [*F*_(1, 36)_ = 1.743, *p* = 0.195, η^2^_ρ_ = 0.046], within group analyses indicated that the AD group benefitted from emotional content, showing increased memory for Phase 2 of the emotional story (*p* = 0.027) compared to the neutral story, which was not seen for Phase 1 (*p* = 0.547) or Phase 3 (*p* = 0.773). In bvFTD and controls, no difference in performance between the neutral and emotional stories was seen on any of the phases (all *p* > 0.05), however, a trend for better memory in Phase 2 of the emotional story was observed in the control group (*p* = 0.082). The main effect of phase was significant [*F*_(2, 65)_ = 7.253, *p* = 0.004, η^2^_ρ_ = 0.168], with more peripheral details remembered from Phase 2 than Phase 1 (*p* = 0.005) and Phase 3 (*p* = 0.012). This effect was driven by the bvFTD group who recalled more peripheral details from Phase 2 than Phase 1 (*p* < 0.001) and Phase 3 (*p* = 0.032), averaged across the two stories. Examination of each story separately confirmed this, with the bvFTD group showing better memory for Phase 2 than Phase 1 for both the neutral (*p* = 0.009) and emotional (*p* = 0.011) stories and a trend for better memory in Phase 2 compared to Phase 3 of the neutral story (*p* = 0.065). In contrast, the AD group remembered more details in Phase 2, compared to Phase 1 (*p* = 0.040) but not Phase 3 (*p* = 1.000), for the emotional story only. For controls, no differences were seen across phases for the neutral or emotional story versions (all *p* > 0.05).

In summary, the AD and bvFTD groups remembered fewer peripheral details of the stories than controls, with no significant difference in number of details recalled between patient groups. Emotional content had an enhancing effect on memory for peripheral details, in AD only. Unexpectedly, a difference in the number of peripheral details recalled across phases was observed, with memory better for the middle phase irrespective of emotional content. As was the case for the combined score the bvFTD group drove this effect.

### Voxel-based morphometry group analyses

#### Patterns of atrophy

Compared with controls, bvFTD patients showed decreased gray matter intensity in bilateral frontal and temporal regions, including the anterior cingulate and orbitofrontal and medial prefrontal cortices, as well as the temporal pole bilaterally. In contrast, AD patients showed decreased gray matter intensity in the right hippocampus, right precuneus, left superior and middle frontal cortex and parietal regions bilaterally (Figure S1).

#### Neural correlates of emotional enhancement of memory

**Figure 4:**
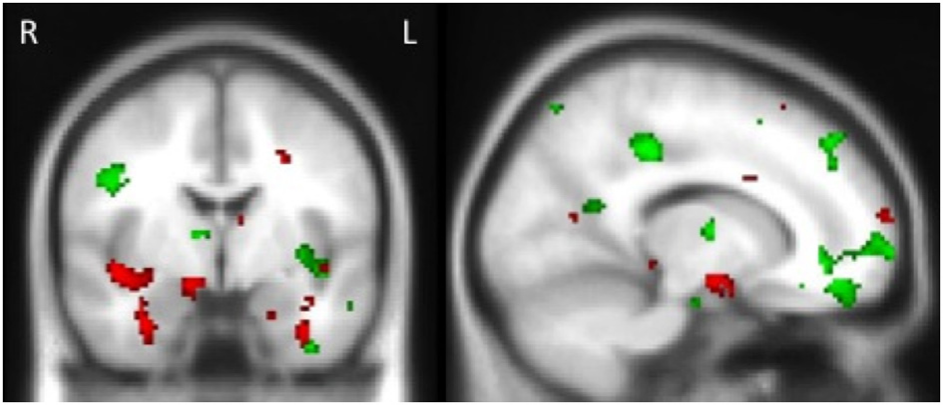
**Voxel-based morphometry results showing regions that correlate with memory performance (green) and emotional enhancement of memory (red) in all participants combined**. Colored voxels show regions that were significant in the analyses *p* < 0.005 uncorrected for multiple comparisons. Clusters are overlaid on the standard Montreal Neurological Institute (MNI) brain. MNI Coordinates: *x* = 14, *y* = −4, *z* = −16.

Examination of the neural correlates supporting memory in all groups combined revealed that reduced gray matter intensity in the left hippocampus, left medial frontal cortex, right posterior cingulate, left temporal pole and left occipital cortex was associated with memory for the stories irrespective of emotional content. In contrast, the emotional enhancement of memory effect was associated with integrity of the right amygdala, right insula and bilateral parahippocampal cortices (Figure 4, Table 4).

**Table 4:** **Voxel-based morphometry results showing regions of gray matter intensity that covary with memory performance and emotional enhancement of memory in all participants combined at *p* < 0.005, uncorrected for multiple comparisons**.

**Regions**	**BA**	**Hemisphere**	**MNI coordinates**			**Number of voxels**
			***X***	***Y***	***Z***	
**TOTAL SCORE—MEMORY**						
Medial frontal cortex, anterior cingulate, frontal pole	10	Left	−10	40	−14	1978
Middle and superior frontal gyrus	8	Left	−26	26	40	529
Lateral occipital cortex (superior division), angular gyrus	39	Left	−46	−64	18	472
Hippocampus extending into putamen, insular cortex, planum polare	28	Left	−34	−14	−10	364
Superior frontal gyrus, paracingulate gyrus	6	Right	2	18	56	305
Inferior frontal gyrus	45	Left	−48	24	16	232
Angular gyrus	39	Right	44	−58	28	190
Angular gyrus, supramarginal gyrus (posterior division)	40	Right	48	−48	36	176
Posterior cingulate	31	Right	10	−32	38	164
Frontal pole	10	Left	−26	48	22	151
Inferior frontal gyrus	9	Left	−34	16	26	115
Post−central gyrus	6	Right	46	−8	28	111
Temporal pole	38	Left	−48	2	−42	102
Lateral occipital cortex (superior division)	19	Left	−30	−82	8	100
**TOTAL SCORE—EMOTIONAL ENHANCEMENT**						
Lateral occipital cortex, occipital pole	18	Left	−46	−84	−8	1125
Temporal fusiform cortex extending into amygdala, parahippocampal cortex, hippocampus	20	Left	−35	−6	−34	1176
Temporal fusiform cortex, parahippocampal cortex, hippocampus, insula	20	Right	32	−16	−34	1045
Frontal pole	10	Left	−34	56	18	929
Frontal pole	10	Right	36	48	34	306
Middle temporal gyrus	21	Right	42	−46	8	206
Frontal pole	10	Right	12	68	14	121
Parietal operculum cortex	13	Right	32	−28	22	112

BA, Brodmann Area.

When we examined the neural correlates of memory for central and peripheral details separately, we found distinct associations depending on the type of detail. Memory for central details irrespective of emotional content, correlated with the frontal pole, lateral occipital cortex, left insula and precentral gyrus, whereas emotional enhancement of memory for central details was associated with the right superior temporal gyrus and insula as well as the hippocampus and parahippocampal gyri bilaterally (Figure S2, Table S1). In contrast, memory for peripheral details irrespective of emotional content was associated with an extensive network of regions in the frontal pole and prefrontal cortex. Emotional enhancement of memory for peripheral details was also correlated with the frontal pole and left hippocampus and parahippocampal gyrus as well as the right middle temporal gyrus (Figure S3, Table S2).

**Figure 5:**
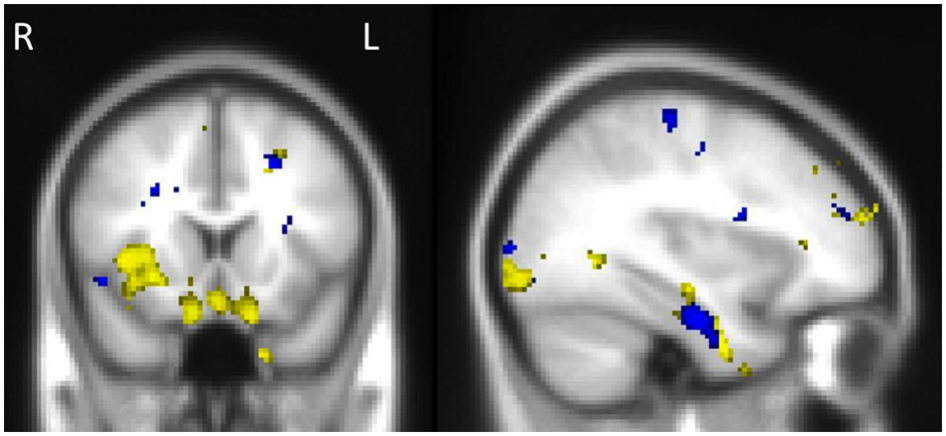
**Voxel-based morphometry results showing regions that correlate with emotional enhancement of memory in bvFTD (yellow) and AD (blue)**. Colored voxels show regions that were significant in the analyses *p* < 0.005 uncorrected for multiple comparisons. Clusters are overlaid on the standard Montreal Neurological Institute (MNI) brain. MNI Coordinates: *x* = −34, *y* = 16, *z* = −22. All clusters reported *t* > 2.80.

Investigation of structures that were associated with emotional enhancement of memory for combined (central and peripheral) memory in bvFTD and AD separately, revealed distinct neural correlates according to diagnostic group. The degree of emotional enhancement of memory in bvFTD was associated with gray matter intensity in the orbitofrontal cortex, right insula, hippocampus, amygdala and temporal fusiform regions. Whereas in AD, emotional enhancement of memory correlated with integrity of a distributed set of regions including the bilateral hippocampus and parahippocampal gyri, temporal fusiform cortex and the frontal pole (Figure 5, Table 5).

**Table 5:** **Voxel-based morphometry results showing regions of significant gray matter intensity decrease that covary with emotional enhancement of memory in bvFTD and AD at *p* < 0.005, uncorrected for multiple comparisons**.

**Regions**	**BA**	**Hemisphere**	**MNI coordinates**			**Number of voxels**
			***X***	***Y***	***Z***	
**bvFTD**						
Middle temporal gyrus (posterior division), temporal fusiform cortex, hippocampus, amygdala, orbitofrontal cortex, insula	20	Right	64	−20	−16	2696
Lateral occipital cortex (superior division)	39	Left	−54	−72	−24	1413
Frontal pole	46	Right	42	48	18	1383
Temporal fusiform cortex (anterior division), parahippocampal gyrus	20	Left	−34	−2	−38	228
Frontal pole	10	Left	−40	60	18	172
Precuneus	30	Right	20	−68	12	144
Superior frontal gyrus	8	Left	−20	30	40	121
**AD**						
Middle frontal gyrus, precentral gyrus	6	Left	−26	0	40	352
Planum temporale	13	Right	32	−28	18	342
Post−central gyrus	4	Left	−36	−26	60	193
Temporal fusiform gyrus, parahippocampal gyrus, hippocampus	20	Left	−36	−14	−26	144
Frontal pole	10	Left	−28	46	24	122
Planum polare, insular cortex	21	Right	44	−2	−10	111
Middle temporal gyrus (anterior division)	21	Left	−64	4	−14	109
Parahippocampal gyrus, hippocampus	20	Right	32	−10	−34	106
Temporal fusiform gyrus (posterior division)	37	Left	−26	−40	−22	100

BA, Brodmann Area.

## Discussion

This study examined emotional enhancement of memory for a naturalistic complex event in bvFTD compared with AD and investigated the impact of emotion on memory for central and peripheral details across these syndromes. Our findings confirm that emotional enhancement of memory is compromised in bvFTD (Kumfor et al., 2013b) and demonstrate for the first time that this deficit extends to memory for detailed scenarios similar to what patients experience in day-to-day life. In contrast, emotion enhanced memory for an engaging ecologically-valid story in mild AD patients. Voxel-based morphometry analyses revealed that emotional enhancement of memory in bvFTD and AD depends on the integrity of brain regions involved in emotion processing including the amygdala, insula, orbitofrontal cortex, and memory such as the hippocampus and frontal pole. Here, we discuss how our findings inform cognitive and neurobiological theories of emotional memory.

At a behavioral level, we demonstrated that memory for ecologically valid emotional events is attenuated in bvFTD. Importantly, this decline is not simply due to differences in how the emotional content of events is perceived, or how well events are subjectively understood. This lack of enhancement is similar to that seen in healthy individuals who have been administered a beta-blocker, which dampens changes in autonomic arousal when viewing an emotional event (Cahill et al., 1994). The few studies measuring physiological responses in bvFTD have demonstrated that arousal to emotional stimuli is indeed compromised in these patients. A single fear conditioning study in bvFTD (in a mixed frontotemporal dementia cohort) with respect to AD (Hoefer et al., 2008) revealed that while both patient groups showed abnormal fear conditioning, the underlying cause differed. In bvFTD, fear conditioning was compromised due to decreased physiological arousal, whereas in AD the deficit was isolated to abnormal conditioning. Additional studies also suggest that arousal during complex emotional situations (e.g., singing karaoke) is dampened in these patients (Sturm et al., 2008). Psychophysiological assessment of emotional reactivity in bvFTD is relatively rare; although our results are consistent with the hypothesis that changes in emotional arousal underlie the loss of emotional enhancement of memory in bvFTD. This will be an important avenue for future studies to address.

Our findings in bvFTD stand in contrast to AD where an emotional enhancement of memory effect was observed, despite an overall reduction in memory performance. This pattern of performance is in line with studies of individuals with amnesia, who show preserved emotional enhancement despite marked memory impairment (Hamann et al., 1997). Previous investigations of emotional enhancement of memory in AD have reported mixed results (e.g., Ikeda et al., 1998; Mori et al., 1999; Kensinger et al., 2004). The disparity across studies appears to be related to differences in patient characteristics and/or methodological approaches (Bennion et al., 2013). The patients included in this study were mild, thus ensuring that floor effects did not mask any facilitatory effect of emotion on memory. In addition, this study used emotionally engaging material, which may be particularly relevant for AD (Ikeda et al., 1998; Kazui et al., 2000; Moayeri et al., 2000). It should be noted that despite emotion enhancing memory in AD, the emotional manipulation did not restore memory in AD to normal limits. Whether this enhancing effect is clinically meaningful remains to be seen. Crucially, here we have demonstrated that emotion has distinct effects on memory in bvFTD and AD, using emotionally engaging, ecologically valid stimuli, in a mild dementia cohort.

### Memory for central and peripheral details

The second aim of this study was to examine trade-off effects in bvFTD and AD. Interestingly, here, we uncovered differential effects of emotion on memory for central and peripheral details across groups. In bvFTD, no effect of emotion was seen on memory for either central or peripheral details. It is possible that the lack of effect of emotion on memory for central details was due to the relatively preserved memory for central details in bvFTD, in line with their relatively preserved recognition memory. While this account cannot be ruled out, the attenuation of emotional enhancement of memory for central, peripheral and combined scores observed here is consistent with the lack of emotional influence on simple visual recognition of emotional stimuli (Kumfor et al., 2013b), and fear conditioning (Hoefer et al., 2008) previously reported in these patients.

In contrast to bvFTD, memory for central details in AD was enhanced for the emotional story compared to the neutral story. This study is the first to tease apart the effects of emotion on central and peripheral details in this patient group and confirms that despite severe episodic memory deficits, emotion impacts on the nature and quality of memories in patients with AD. Interestingly, however, a traditional “trade-off effect” was not seen in AD, with emotion also enhancing memory for peripheral details in these patients. The trade-off effect on memory for central vs. peripheral details is proposed to depend on differential effects of attention, with emotion causing attention to be disproportionately focused on information central to the event. This trade-off is often demonstrated using images where memory for the main object of the picture (the central detail) is assessed in comparison to memory for the background (the peripheral detail) (e.g., Kensinger et al., 2005, 2007). It is possible that the enhancing effect of emotion on memory for some peripheral details is a by-effect of increased attention toward central details of the picture. This effect may be more pronounced in AD because their reduced overall memory allows greater room for improvement. This account is consistent with previous reports of emotional enhancement of memory for central *and* peripheral details, on tasks similar to the one used here (Heuer and Reisberg, 1990). Future studies using stimuli that test memory for central and peripheral details that are not confounded by attention indirectly focused on “peripheral details” are needed to determine whether this position is correct. In addition, clear definitions of these trade-off effects between central/peripheral, intrinsic/extrinsic and gist/detail are needed in order to examine potential trade-off effects of emotion on memory (Kensinger et al., 2007; Kensinger, 2009).

The lack of enhancement for central details in healthy control participants was somewhat unexpected, considering that previous investigations have suggested the greatest effect of emotional enhancement is on memory for details central to the story. Unlike previous studies, this study used a 1-h rather than a 1-week delay, due to the anticipated memory deficits in the patient groups. As a result of this short delay, performance in controls was high (approximately 80% correct). As such, the relative lack of emotional enhancement specific to central details in controls may reflect a ceiling effect for memory of both stories. It is also accepted that the effect of emotion is partly modulated by consolidation effects, with the emotional enhancement effect strengthening over time (Hamann, 2001). It is therefore likely that with an extended delay the *trade-off* effect would be observed in controls. Nevertheless, the pattern of performance is similar to what would be predicted, with a higher number of details recalled on average, for Phase 2 of the emotional than the neutral story in controls.

### Brain regions supporting emotional enhancement of memory

Our study has further revealed that different brain networks are involved in emotional enhancement of memory in bvFTD and AD. Integrity of the orbitofrontal cortex has been associated with emotional memory in bvFTD previously (Kumfor et al., 2013b). The results from this study extend previous findings and demonstrate that the amygdala and insula together with the orbitofrontal cortex, contribute to emotional enhancement of memory for complex realistic events in bvFTD. Previous studies have suggested that two routes to emotional enhancement of memory exist: an amygdala-hippocampal network which is associated with enhancement for arousing stimuli and a prefrontal cortex-hippocampal network which enhances memory for non-arousing, but valenced stimuli (Kensinger and Corkin, 2004; Labar and Cabeza, 2006; Mickley Steinmetz and Kensinger, 2009). While the amygdala dependent network is thought to increase attentional resources and facilitate encoding and consolidation, the prefrontal network is proposed to improve memory through elaborative encoding of emotional stimuli (Kensinger, 2004). The neuroimaging results suggest that both these routes are compromised in bvFTD. While the role of the right amygdala in emotional memory is well established (Cahill et al., 1995, 1996) especially for arousing information, the involvement of the right insula is also of significance given its reported role in processing somatosensory information and interoceptive awareness (Craig, 2009). The insula has been shown to activate during retrieval of emotional memories of individuals with post-traumatic stress disorder (Rauch et al., 1996) and during retrieval of emotionally-laden autobiographical memories (Fink et al., 1996). These results therefore provide additional support to the hypothesis that insufficient emotional arousal may be driving the attenuation of emotional enhancement of memory. Our results further suggest that both abnormal changes in arousal and inefficient controlled encoding processing may contribute to the loss of emotional enhancement of memory in these patients.

In AD, in contrast, the middle frontal gyrus, bilateral hippocampus, parahippocampal gyri and frontal pole were associated with the degree of emotional enhancement, regions which are involved in episodic memory performance (Squire et al., 2004; Ranganath and Ritchey, 2012). These results support previous findings that alterations in memory ability determine the extent that mild AD patients are able to benefit from the emotional content of events to facilitate memory (Kumfor et al., 2013b). Examination of the effect of emotion on memory in AD over time will be important in establishing the effect of emotion on memory in the latter stages of the disease. Notably in AD, integrity of the right insula was also associated with emotional enhancement of memory, suggesting that the ability to identify changes in autonomic arousal and use this information when encoding and retrieving emotional memories may be particularly important for the emotional enhancement of memory to proceed, especially for memory for complex emotional events.

It should be noted that our control group had a higher level of education than our patient groups. Importantly, here we used a within-subjects design to examine the effect of emotion on memory, with each participant acting as their own control. It is therefore unlikely that differences in education account for the differential effects of emotion on memory in bvFTD and AD, compared to healthy older adults. Previous studies in healthy adults have used between-subjects designs in order to rule out the potential effects of asymmetric transfer. This approach was not possible here, given the small sample sizes inherent to clinical studies. Consequently, possible effects of story order cannot be entirely ruled out. Asymmetric transfer, however, would be expected to have similar effects across both the control and patient groups, and therefore unlikely accounts for the differential effects of memory in bvFTD and AD seen here. A final issue to note is that the effect sizes observed were variable. Although clear differential effects of emotion on memory were observed in bvFTD and AD, the extent that these effects are clinically meaningful is yet to be determined, and is an important area for future studies to address.

While our results provide important evidence that emotion does not facilitate memory for negatively valenced emotional events in bvFTD, it is unclear whether this extends to positively valenced emotional events. The literature examining emotional enhancement of memory has focused primarily on negative events, as these highly arousing events produce a reliable and measurable effect on memory. From a clinical perspective, however, whether memory for positive events such as weddings, or the birth of a grandchild, is enhanced in these patients has clear implications. The proposal that valenced but non-arousing events rely on elaborative encoding via the prefrontal cortex (Kensinger and Corkin, 2004; Mickley and Kensinger, 2008; Mickley Steinmetz and Kensinger, 2009) is of particular relevance for emotional enhancement of positive life events such as those outlined above. BvFTD patients offer an opportunity to examine these two routes of emotional enhancement of memory and to investigate how emotion facilitates memory for both negative and positive events.

To summarize, this study has revealed an important dissociation between the effect of emotion on memory in bvFTD and AD. While bvFTD patients showed no appreciable effects of emotion on memory, AD patients exhibited an enhancing effect of emotion on overall memory, as well as on memory for central and peripheral details. The results suggest that integrity of systems involved in emotion processing, especially emotional arousal, such as the orbitofrontal cortex, amygdala and insula are crucial for emotional enhancement of memory. Conversely, these findings reinforce existing evidence that emotion can positively influence memory, even in individuals where episodic memory ability is compromised. This study is the first to examine how emotion impacts on memory for complex events in bvFTD and suggests that changes in arousal may contribute to the attenuation of emotional enhancement of memory in these patients. Measuring arousal levels in response to emotional stimuli in these patients will be an important next step for future studies to address.

## Author contributions

Fiona Kumfor was responsible for the design of the study, data acquisition and analysis, interpretation and manuscript preparation. Muireann Irish was responsible for data analysis and interpretation and manuscript preparation. John R. Hodges was responsible for the design of the study, data interpretation and manuscript preparation. Olivier Piguet was responsible for the design of the study, data analysis and interpretation and manuscript preparation.

### Conflict of interest statement

The authors declare that the research was conducted in the absence of any commercial or financial relationships that could be construed as a potential conflict of interest.
