# Supplementary material for: Frontal and temporal lobe contributions to emotional enhancement of memory in behavioral-variant frontotemporal dementia and Alzheimer's disease
Source: Front Behav Neurosci. 2014 Jun 24;8:225. doi: 10.3389/fnbeh.2014.00225 (PMC4067999; doi:10.3389/fnbeh.2014.00225)
Supplement: Supplementary file 1 [file DataSheet1.DOCX]

**Supplementary Material**

*
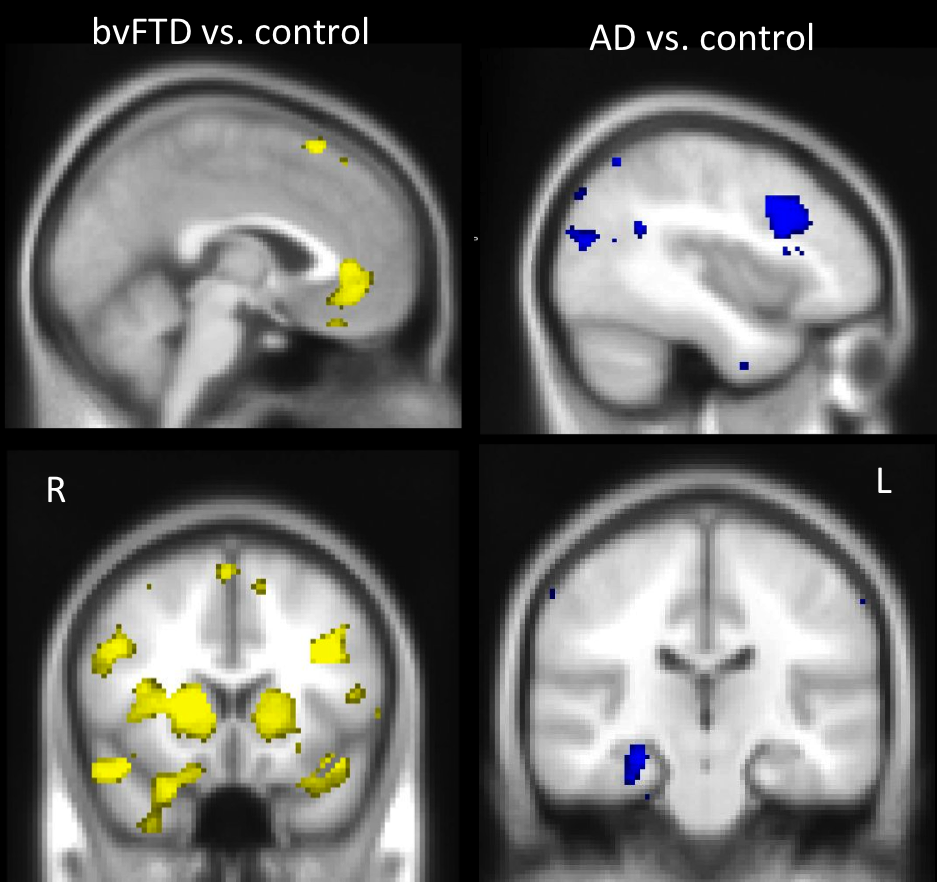
*

*Supplementary Figure 1.* Regions of atrophy in bvFTD and AD

*Note.* Voxel-based morphometry results showing regions where grey matter intensity was lower in (i) bvFTD compared to controls x = -2, y = 16, z = -14; and (ii) AD compared to controls x = -40, y = -22, z = -18. Coloured voxels show regions significant in the analyses with *p* < .005 uncorrected for multiple comparisons. Clusters are overlaid on the standard Montreal Neurological Institute (MNI) brain. R = right, L = left.

***
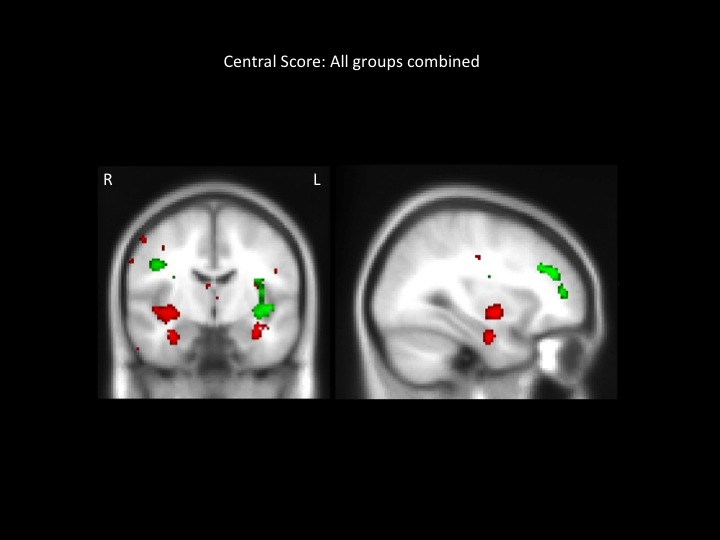
***

***Supplementary Figure 2.*** Voxel-based morphometry results showing regions that correlate with memory performance (green) and emotional enhancement of memory (red) for Central details in all participants combined. *Note.* Coloured voxels show regions that were significant in the analyses *p* < .005 uncorrected for multiple comparisons. Clusters are overlaid on the standard Montreal Neurological Institute (MNI) brain. MNI Coordinates: x = 32, y = -8, z = -26.

***Supplementary Table 1.*** Voxel based morphometry showing significant correlations between grey matter intensity and memory for central details in all participants combined at *p* < .005, uncorrected for multiple comparisons.

| **Regions** | **BA** | **Hemisphere** | **MNI coordinates** | | | **Number of voxels** |
| --- | --- | --- | --- | --- | --- | --- |
|  |  |  | **X** | **Y** | **Z** |  |
| **Central Score - Memory** |  |  |  |  |  |  |
| Frontal pole | 10 | Right | 18 | 70 | -2 | 540 |
| Insular cortex, putamen | 13 | Left | -42 | -10 | -4 | 534 |
| Lateral occipital cortex | 19 | Left | -36 | -78 | 6 | 392 |
| Precentral gyrus | 6 | Right | 44 | -2 | 26 | 140 |
| **Central Score – Emotional Enhancement** |  |  |  |  |  |  |
| Superior temporal gyrus, insular cortex, planum polare | 21 | Right | 58 | 2 | -8 | 724 |
| Temporal pole | 38 | Left | -32 | 12 | -48 | 610 |
| Parahippocampal gyrus (posterior division), thalamus | 28 | Right | 14 | -34 | -8 | 497 |
| Thalamus | 20 | Left | -18 | -24 | -4 | 404 |
| Cerebellum | - | Left | -10 | -82 | -36 | 380 |
| Hippocampus | 34 | Right | 34 | -8 | -24 | 318 |
| Lateral occipital cortex | 37 | Left | -58 | -68 | 8 | 287 |
| Hippocampus | 34 | Left | -32 | -12 | -22 | 190 |
| Postcentral gyrus | 4 | Right | 46 | -16 | 44 | 140 |
| Cerebellum | - | Right | 12 | -58 | -60 | 125 |

*Note.* BA = Brodmann area.


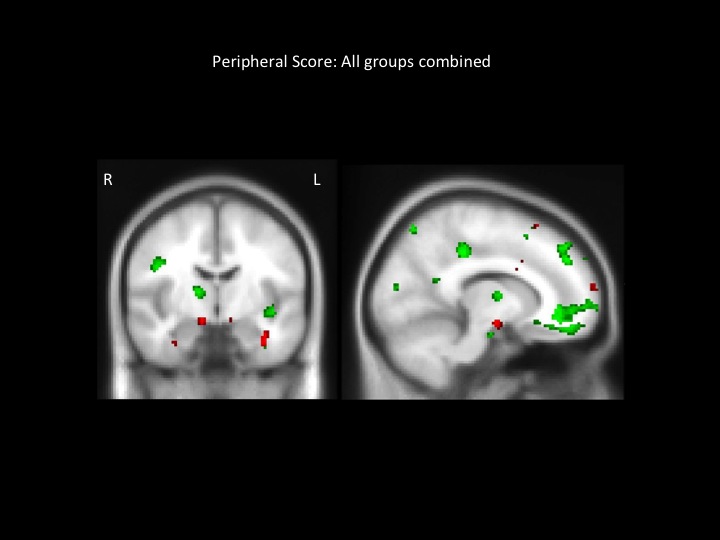


***Supplementary Figure 3.*** Voxel-based morphometry results showing regions that correlate with memory performance (green) and emotional enhancement of memory (red) for Peripheral details in all participants combined. *Note.* Coloured voxels show regions that were significant in the analyses *p* < .005 uncorrected for multiple comparisons. Clusters are overlaid on the standard Montreal Neurological Institute (MNI) brain. MNI Coordinates: x = 12, y = -8, z = -14.

***Supplementary Table 2.*** Voxel based morphometry showing significant correlations between grey matter intensity and memory for peripheral details in all participants combined at *p* < .005, uncorrected for multiple comparisons.

| **Regions** | **BA** | **Hemisphere** | **MNI coordinates** | | | **Number of voxels** |
| --- | --- | --- | --- | --- | --- | --- |
|  |  |  | **X** | **Y** | **Z** |  |
| **Peripheral Score - Memory** |  |  |  |  |  |  |
| Inferior frontal gyrus, middle frontal gyrus, superior frontal gyrus | 45 | Left | -48 | 24 | 16 | 1590 |
| Frontal pole, medial frontal cortex, anterior cingulate | 11 | Right | 14 | 44 | -16 | 1324 |
| Frontal pole, inferior frontal gyrus, middle frontal gyrus | 46 | Right | 42 | 34 | 12 | 708 |
| Lateral occipital cortex (anterior division) | 39 | Left | -46 | -62 | 22 | 530 |
| Supramarginal gyrus (anterior division) | 40 | Right | 48 | -44 | 34 | 249 |
| Temporal pole | 38 | Left | -48 | 2 | -42 | 219 |
| Insula | 21 | Left | -36 | -12 | -10 | 171 |
| Middle temporal gyrus | 21 | Right | 54 | -16 | -12 | 159 |
| Posterior cingulate | 31 | Right | 12 | -34 | 40 | 155 |
| Frontal pole | 10 | Left | -28 | 58 | 20 | 148 |
| Lateral occipital cortex (superior division) | 39 | Right | 54 | -76 | 22 | 139 |
| Superior frontal gyrus | 6 | Right | 18 | 8 | 52 | 100 |
| **Peripheral Score – Emotional Enhancement** |  |  |  |  |  |  |
| Frontal pole | 10 | Right | -30 | 46 | 24 | 807 |
| Middle temporal gyrus (posterior division) | 20 | Right | 48 | -28 | -12 | 281 |
| Parahippocampal gyrus (anterior division), hippocampus | 20 | Left | -34 | -14 | -28 | 266 |
| Lateral occipital cortex (superior division) | 39 | Right | 38 | -58 | 26 | 213 |

*Note.* BA= Brodmann area
